# Preparation and Characterization of Water-Soluble Xylan Ethers

**DOI:** 10.3390/polym9040129

**Published:** 2017-03-31

**Authors:** Kay Hettrich, Ulrich Drechsler, Fritz Loth, Bert Volkert

**Affiliations:** 1 Fraunhofer Institute for Applied Polymer Research IAP, Geiselbergstr. 69, 14476 Potsdam-Golm, Germany; fritz.loth@gmx.de (F.L.); bert.volkert@iap.fraunhofer.de (B.V.); 2 Salzenbrodt GmbH & Co KG, Hermsdorfer Str. 70, D-13437 Berlin, Germany; Ulrich.Drechsler@collonil.de

**Keywords:** hemicellulose, xylan ether, solubility, etherification

## Abstract

Xylan is a predominant hemicellulose component that is found in plants and in some algae. This polysaccharide is made from units of xylose (a pentose sugar). One promising source of xylan is oat spelt. This feedstock was used for the synthesis of two xylan ethers. To achieve water soluble products, we prepared dihydroxypropyl xylan as a non-ionic ether on the one hand, and carboxymethyl xylan as an ionic derivative on the other hand. Different preparation methods like heterogeneous, pseudo-homogeneous, and homogeneous syntheses were compared. In the case of dihydroxypropyl xylan, the synthesis method did not significantly affect the degree of substitution (DS). In contrast, in the case of carboxymethyl xylan, clear differences of the DS values were found in dependence on the synthesis method. Xylan ethers with DS values of >1 could be obtained, which mostly show good water solubility. The synthesized ionic, as well as non-ionic, xylan ethers were soluble in water, even though the aqueous solutions showed slight turbidity. Nevertheless, stable, transparent, and stainable films could be prepared from aqueous solutions from carboxymethyl xylans.

## 1. Introduction

Native polysaccharides such as cellulose, starch, and most of hemicelluloses are not soluble in cold water without preceding chemical modification. To make them soluble in water, polysaccharide ethers and esters are produced. Such polysaccharide ethers are widely used in everyday water-based applications. Typical examples are carboxymethyl cellulose (CMC), which is the most-used cellulose ether; methyl cellulose (MC), hydroxyethyl cellulose (HEC) and hydroxypropyl cellulose (HPC), as well as cationic starches, or hydroxyethyl starch (HES) and hydroxypropyl starch (HPS). Cellulose and starch ethers are used as film formers, protective colloids, stabilizers, thickening agents, adhesives or detergent additives in the food, pharmacy, paper and textile industries, as well as in the building and mining industry.

Next to cellulose, hemicellulose is the second most abundant class of polysaccharides found in nature. It is the third key structural ingredient of the wooden cell-wall, besides cellulose and lignin. Furthermore, hemicellulose is found in vegetable fibers and in cell-walls of grass and corn. Thus, hemicellulose is an attractive raw renewable resource for new biobased materials and functional polymers that can be adapted by chemical reactions. In contrast to cellulose, hemicellulose is a non-crystalline heteropolysaccharide, which can be extracted from plant or wood by water and/or aqueous alkali [1,2,3,4,5]. Depending on the phylogeny, hemicellulose can be comprised of glucans, xyloglucans, xylans, arabinoxylans, mannans, gluco- or galactomannan with different substitution patterns [6]. Accordingly, hemicellulose may contain C_5_ or C_6_ sugar units. 

**Figure 1:**
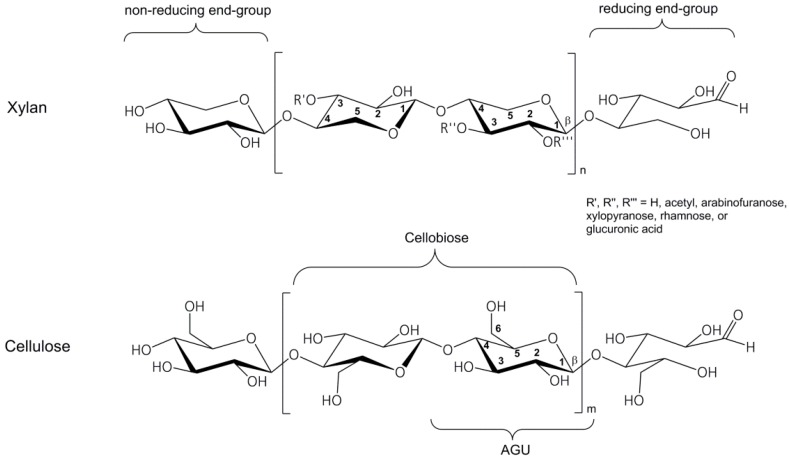
Structure of a xylan from annual plant [9] in comparison to cellulose; in both polymers the monomer units are linked together by β-(1 → 4) glycosidic bonds; AGU = anhydroglucose unit.

Xylans, for example, are a prevalent hemicellulose component found in plants and in some algae, which are made from units of xylose (C_5_ sugar units) (Figure 1). Xylans compose about 10–15% of softwoods, about 10–35% of hardwoods and 35–40% of the total mass in the residues of annual plants, e.g., in husks or oat spelt, which are the residues of oat flake production. Xylan shows characteristic differences in comparison with cellulose or starch, such as a lower degree of polymerization (DP); this means lower chain length and lower molar masses, and the absence of the primary OH-group in position C6. Note that, in the case of C_6_ polysaccharides, the primary OH-group at position C-6 in the anhydroglucose unit (AGU) is the most reactive group in comparison with the two secondary OH-groups at position C-2 and C-3. Therefore, the aim of this study was to explore if it is possible to modify xylan chemically by methods comparable to those that are established for the modification of polysaccharides at an industrial scale, on the one hand; and to achieve water soluble xylan products similar to cellulose or starch derivatives, on the other hand.

The structure of xylan varies depending on its herbal origin. The different structures have been summarized in several reviews on hemicelluloses in wood [7,8], grass [9], and cereals [10,11]. Some aspects about the structure of xylan from oat spelts were discussed by Saake et al. [12]. Oat spelts contain high amounts of xylan and have relatively low lignin content. Hence, this raw material is an interesting source for the isolation of xylan. A proposal for the structure of xylan in annual plants is shown in Figure 1 [9]. The monomer units are linked together by β-(1 → 4) glycosidic bonds. Typical for xylan is the presence of side chains made of arabinofuranose, xylopyranose, rhamnose, glucuronic acid, and acetyl groups. Most of these sugar residues are connected by acetal bonds, while the acetyl groups are connected by ester bonds. Typical degree of substitution (DS) values of these side groups are between 0.02 and 0.2 in the case of arabinose, between 0.02 and 0.08 of glucuronic acid, <0.02 of all other sugar units, and between 0.02 and 0.15 of acetyl groups [13]. Usually, the content of acetyl groups in extracted xylan is much lower because the ester bonds are cleaved during the alkaline extraction.

Despite promising application possibilities, xylan derivatives have not been used in quantities large enough for industrial processing up until now. Because of the possibility of isolating xylan from residues of annual plants, it is a particularly cheap biobased raw material which can be used for different applications. A typical example is oat spelt, which up until now has been mostly used for generation of energy. While they have been also used as additives for animal feed similar to cereal straw, the EU Food Safety Regulation 178/2002 makes it increasingly difficult for the food industry to deliver mill products, such as oat spelt, to the animal feed industry. Hence, oat spelt is widely available for material utilization, with approximately 150,000 t of oat spelt occurring just in Germany in 2014 [14]; their use avoids the conflict of food vs. raw material for chemical industry. 

Xylans with a low DP of approximately <30 are water soluble. For the preparation of water soluble xylan with a higher DP, derivatization is necessary, e.g., by incorporation of hydrophilic groups. High DP values are, in turn, necessary for some interesting applications, such as film forming. Hence, the wish to replace petrochemical-based plastics—for example, replacing food packaging films with biodegradable ones—motivates the study of hemicellulose derivatives and their properties. 

Previously, the gel- and film-forming properties of xylans were investigated by several studies. For instance, the nature of oxidative cross-linking of arabinoxylan was shown by Ng et al. [15]. The film-forming properties were examined for various mixtures of this hemicellulose and chitosan [1,16]. The stability of the films can be influenced by addition of various plasticizers, such as propylene glycol, glycerol, or sorbitol, in concentrations up to 22% in an arabinoxylan solution [17].

The synthesis of hemicellulose methyl ether was described by Fang et al. [18]. The ether was prepared by methylation with methyl iodide in dimethyl sulfoxide, using sodium hydride as a catalyst. However, different from methylcellulose, the described hemicellulose ethers were not soluble in water.

A detailed report on the extraction of xylanes, their chemical modification, and their applicability, was given by Ebringerova and Hromadkova [19]. Water soluble amphiphilic derivatives of beech wood xylan and its sulfoethyl derivatives were obtained by the introduction of low amounts of long alkyl chains. The derivatives showed excellent emulsifying and foam-stabilizing properties. The synthesis of water soluble xylan sulfate ester has been reported in detail in a previous paper [20]. The preparation of the xylan sulfates succeeded at a broad DS range, from 0.58 to 1.95, by controlling the synthesis parameters. Other reviews summarize the chemical functionalization of xylan by different authors [20,21,22]. Nypelö and coworkers specified the synthesis of hydroxypropyl and 3-butoxy-2-hydroxypropyl xylan recently, with DS values in the range of 0.28–0.73 and 0.2–1.0, respectively [23]. In the case of the hydroxypropyl xylan, water solubility increased with higher DS values. More hydrophobic derivatives were obtained by synthesis of 3-butoxy-2-hydroxypropyl xylan. Both xylan derivatives can be used as interfacial modifiers in oil-in-water emulsions.

The derivatization of xylan can be carried out under typically heterogeneous conditions, similar to those associated with most of the cellulose derivatives; or under homogeneous ones, because xylan is soluble in alkaline conditions. The homogenous method implies derivatization after dissolution in a suitable solvent. In contrast, the reaction occurs in slurry without dissolution of the polymer in a heterogeneous synthesis. These two synthesis strategies were compared to a synthesis under pseudo homogeneous conditions in a kneader with the reaction as an emulsion.

The focus of this study is the preparation of carboxymethyl and novel xylan dihydroxypropyl ethers by these different methods. For all the derivatives, their water solubility behavior and their film-forming properties were examined. Fundamentally, it is interesting to discover how the absence of the primary OH-group will influence the reactivity of xylan and the water solution behavior of xylan ethers. Different synthesis possibilities of xylan ethers were thus investigated regarding the potential for future industrial-oriented procedures. Also, the properties of the synthesized xylan ethers, as typical C_5_ polysaccharide representatives, are discussed in comparison with the properties of cellulose and starch ethers. 

## 2. Materials and Methods

Xylan with a molar mass of about 30,000 g/mol, which was isolated by alkaline extraction of oat spelts, was kindly provided by Peter Koelln KGaA, Elmshorn, Germany. A detailed description of the procedures for the isolation and the determination of the molar mass are given in the paper of Puls et al. [24]. The reactants for the modification of xylans and the solvents used for product purification were obtained from Sigma Aldrich (Taufkirchen, Germany) with puriss grade and used as received.

Subsequently, a typical example of the reaction procedure is described for every synthesis variant. 

### 2.1. Synthesis of Dihydroxypropyl Xylan

#### 2.1.1. Variant 1A—Pseudo Homogeneous Synthesis in a Kneader

The synthesis was carried out in a temperate Duplex kneader of the company IKA (Staufen, Germany). A dispersion of 39.6 g of xylan (0.30 mol, 35%, *w*/*w*) in 73.5 g of a 4% NaOH solution (*w*/*w*) was prepared at 25 °C, and homogenized in the kneader for 1 h. After increasing the temperature to 60 °C, 44.5 g (0.60 mol) of 2,3-epoxy-1-propanol was added, and mixing was continued for 4 h. After cooling to room temperature, 50 mL of methanol was added and a pasty slurry was yielded. The material was transferred to a beaker. Adding 200 mL of methanol, the mixture was homogenized by treatment with an Ultra Turax at 12,500 r.p.m. for 15 min. Finally, the pH was adjusted to 8 with 50% acetic acid-methanol mixture (*v*/*v*). After filtration and washing with methanol, the solid product was dried at 60 °C and 75 mbar.

#### 2.1.2. Variant 2A—Reaction as Emulsion in 2-Propanol or in Cyclohexanone

The synthesis was carried out in a 100 mL three-neck flask with dropping funnel, reflux condenser and KPG stirrer. Firstly, 3.3 g (25 mmol) of xylan was dispersed in 51.7 g of 4% NaOH solution (*w*/*w*) and stirred for 18 h at 25 °C. The emulsion was prepared by addition of 2-propanol (or of cyclohexanone) in ratio 1 to 1 in relation to the xylan dispersion (*v*/*v*) during strong stirring. Then, 3.3 mL (50 mmol) of epoxy propanol was dropped into the emulsion. The mixture was stirred for 4 h at 60 °C. Then 400 mL of methanol was added to the mixture, which was homogenized by treatment with an Ultra Turrax at 12.500 r.p.m. for 15 min. Finally, the pH was adjusted to 8 with 50% acetic acid-methanol mixture (*v*/*v*). After filtration and washing with methanol, the solid product was dried at 60 °C and 75 mbar.

#### 2.1.3. Variant 3A—Homogeneous Syntheses

The synthesis was carried out in a 100 mL flask with stirrer and dropping funnel. Firstly, 3.3 g (25 mmol) of xylan was dispersed in 84 g of 4% NaOH solution (*w*/*w*) for 24 h at room temperature. Then, 3.3 mL (50 mmol) of epoxy propanol was dropped into the solution. The mixture was stirred for 24 h at room temperature, before it was precipitated into 400 mL of methanol. Finally, the pH was adjusted to 8 with 50% acetic acid-methanol mixture (*v*/*v*) and after filtration and washing with methanol, the solid product was dried at 60 °C and 75 mbar.

### 2.2. Syntheses of Carboxymethyl Xylan

#### 2.2.1. Variant 1B—Pseudo Homogeneous Synthesis in a Kneader

A dispersion of 13.2 g (100 mmol) of xylan in 73.4 g of 27% NaOH solution (*w*/*w*) was prepared at 25 °C. The dispersion was homogenized in a temperature-controlled Duplex kneader of the company IKA for 18 h. Then, 18.9 g (200 mmol) of monochloroacetic acid (MCA) was added. After increasing the temperature to 60 °C, the mixture was kneaded for 4 h. The temperature was cooled down to room temperature and 50 mL methanol was added, where a pasty material resulted. The slurry was transferred to a beaker. After further addition of 150 mL methanol, the mixture was homogenized by treatment with Ultra Turrax at 12,500 r.p.m. for 15 min. Then, the pH was adjusted to 8 with 50% acetic acid-methanol mixture (*v*/*v*). After filtration, the solid product was washed with a mixture of methanol–water (80/20, *v*/*v*) until the silver nitrate test became negative. Finally, the product was washed with methanol and dried at 60 °C and 75 mbar.

#### 2.2.2. Variant 2B—Reaction as Emulsion in 2-Propanol or in Cyclohexanone

The synthesis was carried out in a 100 mL three-neck flask with dropping funnel, reflux condenser and KPG stirrer. Firstly, 3.3 g (25 mmol) of xylan was dispersed in 51.7 g of 18% NaOH solution (*w*/*w*) and stirred for 18 h at 25 °C. The emulsion was prepared by addition of 2-propanol (or of cyclohexanone) in ratio 1 to 1 in relation of the xylan dispersion (*v*/*v*) during strong stirring. Then, 4.7 g (50 mmol) of solid mono chloroacetic acid (MCA) was added into the emulsion. The mixture was stirred for 4 h at 70 °C. Afterwards, the pH was adjusted to 8 with 50% acetic acid-methanol mixture (*v*/*v*). After filtration, the solid product was washed with a mixture of methanol–water (80/20, *v*/*v*) until the silver nitrate test became negative. Finally, the product was washed with methanol and dried at 60 °C and 75 mbar.

#### 2.2.3. Variant 3B—Homogeneous Synthesis

The synthesis was carried out in a 100 mL flask with stirrer and dropping funnel. Firstly, 3.3 g (25 mmol) of xylan was dispersed in 29.2 g of 10% NaOH solution (*w*/*w*) for 24 h at room temperature. The reaction was started by dropping 3.3 mL (50 mmol) of epoxy propanol into the solution. Then, 1.5 g (37 mmol) of NaOH as 45% aqueous solution was added. After 30 min, 4.7 g (50 mmol) of monochloroacetic acid (MCA) as an aqueous solution (80%, *w*/*w*) was added via a dropping funnel. The mixture was stirred for 72 h at room temperature. The reaction was finished by precipitation into 400 mL of methanol. Then, the pH was adjusted to 8 with 50% acetic acid-methanol mixture (*v*/*v*). After filtration, the solid product was washed with a mixture of methanol–water (80/20, *v*/*v*) until the silver nitrate test became negative. Finally, the product was washed with methanol and dried at 60 °C and 75 mbar.

#### 2.2.4. Variant 4B—Heterogeneous Method (Slurry)

The synthesis was carried out in a cylindrical double coat flask with reflux condenser, dropping funnel and KPG stirrer. Firstly, 3.3 g (25 mmol) of xylan was dispersed in 40 mL of 2-propanol. Then, 1.0 g (25 mmol) of solid NaOH was added to the mixture and stirred intensively. After 10 min, 5.33 g of 45% NaOH solution was added and stirred for 2 h at room temperature. Then, 1.0 g (25 mmol) of solid NaOH and 4.7 g (50 mmol) of solid mono chloroacetic acid (MCA) was added. The mixture was stirred for 4 h at 50 °C. After cooling to room temperature, the pH was adjusted to 8 with a 50% acetic acid-methanol mixture (*v*/*v*). The raw product was filtered and washed with methanol–water (80/20, *v*/*v*) until the silver nitrate test became negative. Finally, the product was washed with methanol and dried at 60 °C and 75 mbar.

### 2.3. Measurements

#### 2.3.1. Solid State ^13^C-NMR Spectroscopy for the Determination of the Dihydroxypropyl Content

^13^C-NMR spectra were recorded by a Varian UNITY 400 NMR spectrophotometer (frequency 100.58 MHz, Varian, Darmstadt, Germany). High-resolution solid-state spectra were recorded with the cross-polarization/magic angle spinning (CP/MAS) method with spinning frequencies of 5–6 kHz. The contact time was 1–2 ms and the repetition time of the experiments was 3 s. For ^13^C-CP/MAS NMR measurements, the samples, which were wetted with lye, were mechanically squeezed. About 0.1 cm^3^ of the xylan derivatives were filled into a sample rotor. Depending on the sample and the specific objective, the measuring time ranged from 1 to 15 h. For the calculation of the DS values of dihydroxypropyl xylans from ^13^C-NMR spectra, the intensity of the signals from the CH_2_ groups (60 ppm to 67 ppm) was used. At first, the whole spectrum was normalized by setting the integral of C-1 signal (98 to 107 ppm) equal to 1. Then, the integral of the superposed CH_2_ signal of C-5 of xylan and CH_2_–OH group from the substituent introduced (at 63 ppm) was corrected accordingly, to yield the intensity due to the substituent in order to obtain the DS. The relative error of the determination is in the range of ±0.05.

#### 2.3.2. Determination of the Carboxymethyl Content by ICP-OES

The sodium content was determined by ICP-OES (inductive coupled plasma—optical emission spectrometry, Perkin Elmer, Rodgau, Germany) after a nitric acid digestion by assisted microwave irradiation. The analysis was performed as double determination. The reproducibility was better than 1%. The degree of substitution (DS) was calculated as follows [25]:

(1) $$\mathrm{DS}=\frac{132\times \%\mathrm{Na}}{2300-80\times \%\mathrm{Na}}$$

#### 2.3.3. Solubility

The solubility of the xylan derivatives was characterized by preparing aqueous solutions (2%, *w*/*w*) and stirring for 18 h at room temperature. The solution turbidity was measured with a nephelometer 2100AN (Hach, Loveland, CO, USA). The calibration of the instrument was carried out with a formazin standard.

## 3. Results and Discussion

The aim of the investigation was to synthesize water soluble xylan derivatives by etherification with an ionic and with a non-ionic reagent. In contrast to the derivatization of cellulose or starch, the more reactive primary group at position C-6 is absent in the case of xylan, and there are only two secondary OH-groups in position C-2 and C-3 which can be substituted. Thus, the maximum DS value which can be obtained is 2. In this study, two possibilities of etherification to introduce functional groups into xylan were examined. On the one hand, we investigated the incorporation of an additional OH-group by using epoxy propanol as reagent. On the other hand, we investigated the incorporation of an anionic group by using chloro acetic acid. The synthesis and the water solving properties, as well as film forming properties, of dihydroxypropyl xylan and carboxymethyl xylan are described below. 

### 3.1. Synthesis of Dihydroxypropyl Xylan

**Figure 2:**
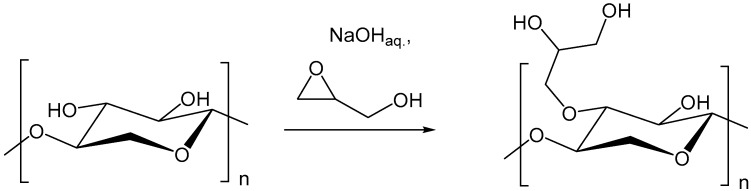
Reaction scheme of the preparation of dihydroxypropyl xylan.

**Figure 3:**
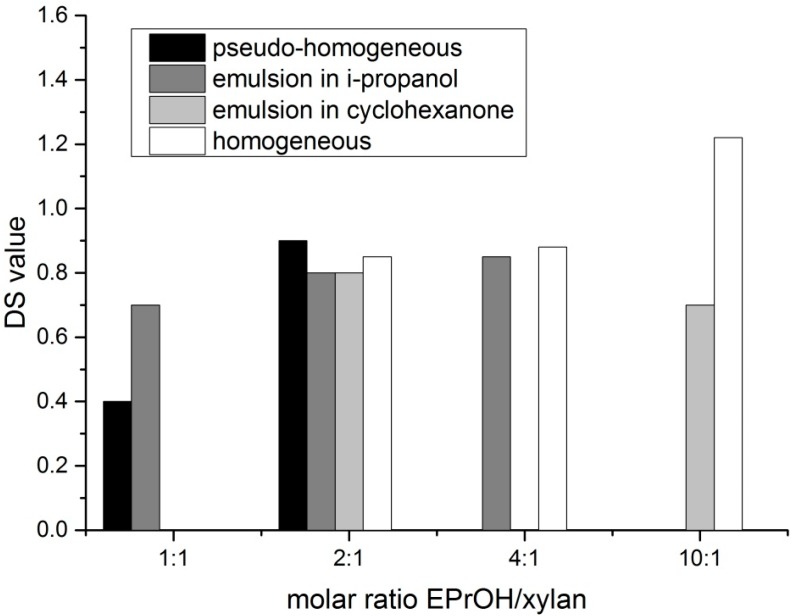
Effect of the reaction conditions on the degree of substitution (DS) values of xylan, achieved with 2,3-epoxy-1-propanol.

**Figure 4:**
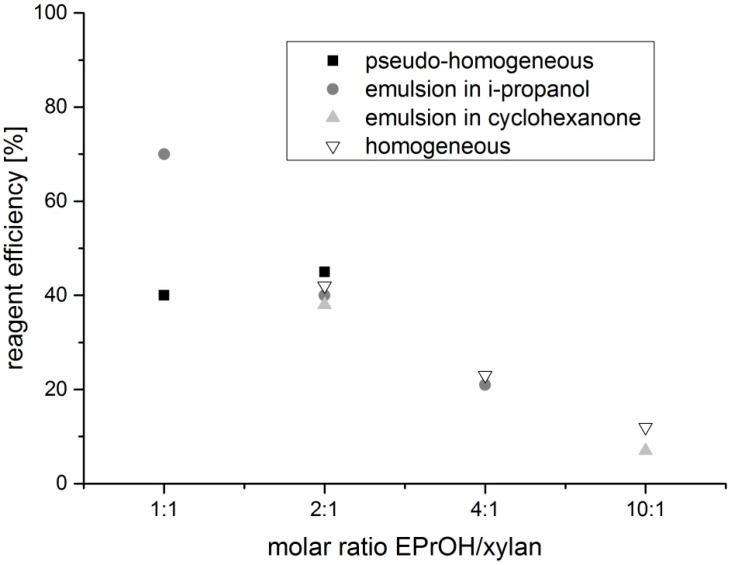
Reagent efficiency in dependence on the amount of epoxypropanol engaged for the various methods used.

The advantage of the preparation of dihydroxypropyl xylan, in comparison to hydroxypropyl xylan, is an extra hydroxyl group, which is incorporated by using epoxy propanol (glycidol) instead of epoxypropan. By using this reagent, a new type of xylan ether is prepared. Additionally, the used etherification reagent is easier to handle because of its higher boiling point. The synthesis of dihydroxypropyl xylan is carried out according to the scheme in Figure 2. The molar ratio of *n*_2,3 epoxy-1-propanol_/*n*_xylan_ as well as ratio of *n*_NaOH_/*n*_xylan_ was changed by using different methods of preparation. Due to the poor water solubility of the dihydroxypropyl xylan prepared under heterogeneous standard slurry conditions, the following derivatives were prepared under more homogeneous conditions, because a more uniform distribution of the substituents along the polymer chain was expected. It is known that the solubility of polysaccharide derivatives is improved when the substituent distribution is more uniform. The results of the various syntheses are shown in Table 1. Dihydroxylpropyl xylans with DS values between 0.4 and 1.2 were obtained. Figure 3 provides an overview of the DS values. Only small differences were observed for the DS values in dependence on the used synthesis method. A DS value of 0.7 was obtained already at a molar ratio *n*_2,3 epoxy-1-propanol_/*n*_xylan_ = 1:1 by using the emulsion method in i-propanol (DHPX4). Surprisingly, a further increase of the reagent 2,3-epoxy-1-propanol, e.g., a molar ratio *n*_2,3 epoxy-1-propanol_/*n*_xylan_ = 4:1, did not lead to a significantly higher DS value. The use of cyclohexanone instead of i-propanol as the emulsion medium did not improve the extent of substitution either. Even the use of a high excess of reagent (*n*_2,3 epoxy-1-propanol_/*n*_xylan_= 10:1; DHPX8) yielded virtually the same DS value. Only under homogeneous conditions, this high excess of epoxy-1-propanol did result in DS values >1 (DPHX12). A clear tendency regarding the influence of the sodium hydroxide concentration under pseudo-homogeneous conditions cannot be recognized (DPHX2 in comparison to DHPX3, in Table 1). The reagent efficiency varied depending on the amount of used reagent; the higher the excess of reagent, the lower is the reagent efficiency (Figure 4). Surprisingly, the reagent efficiency did not show any clear difference with regard to the used preparation method.

**Figure 5:**
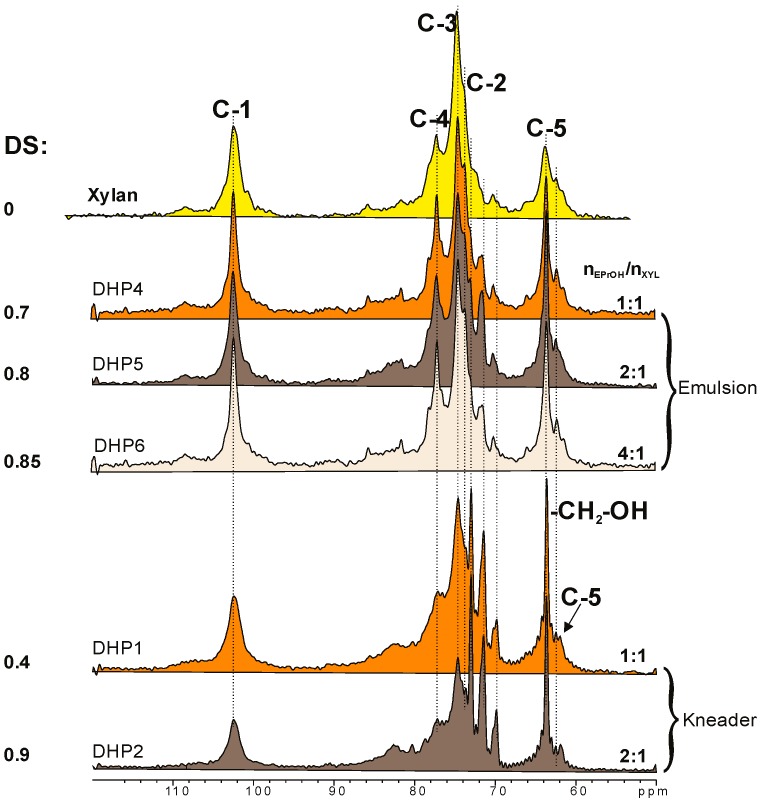
Solid-state ^13^C-NMR (CP/MAS) spectra of selected dihydroxypropyl xylans.

Figure 5 depicts ^13^C-NMR (CP/MAS) spectra of the dihydroxypropyl xylans in comparison with unsubstituted xylan. In general, three characteristic changes in the spectra are seen with increasing DS: first, two rather narrow signals at 70 and 72 ppm emerge due to a shift of the C-2 signal and to the newly formed CH-OH group of the substituent. Second, the peak of the C-5 signal shifts from 64 to 62 ppm. Third, a new signal due to the CH_2_–OH group of the glycerol substituent is seen at 64 ppm. 

Noteworthy is the fact that all products in Table 1 showed good solubility in water. Still, a dependence on the preparation method is observed. The more homogeneous the reaction conditions, the more soluble are the products. 

### 3.2. Syntheses of Carboxymethyl Xylan

**Figure 6:**
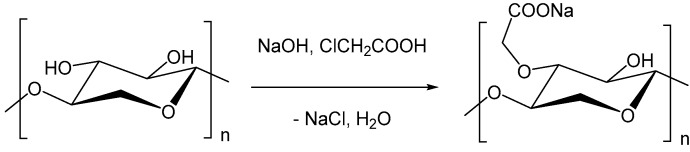
Reaction scheme of the preparation of carboxymethyl xylan.

**Figure 7:**
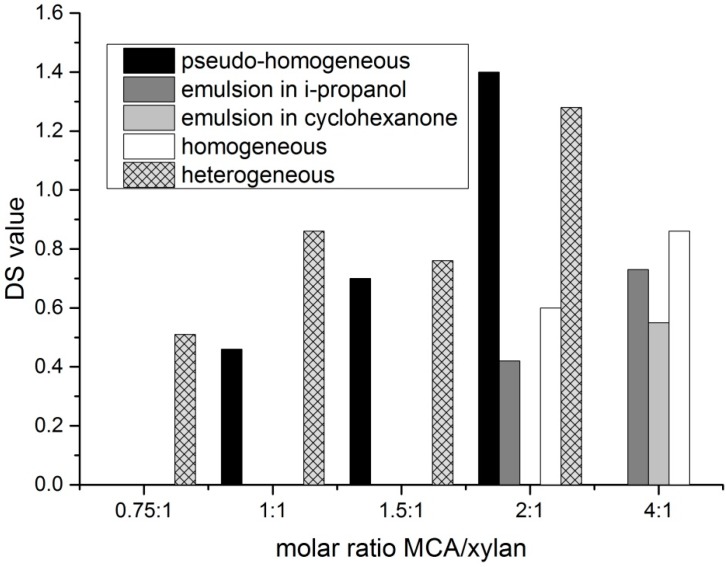
Effect of the reaction conditions on the DS values of xylan, achieved with monochloro acetic acid (MCA).

**Figure 8:**
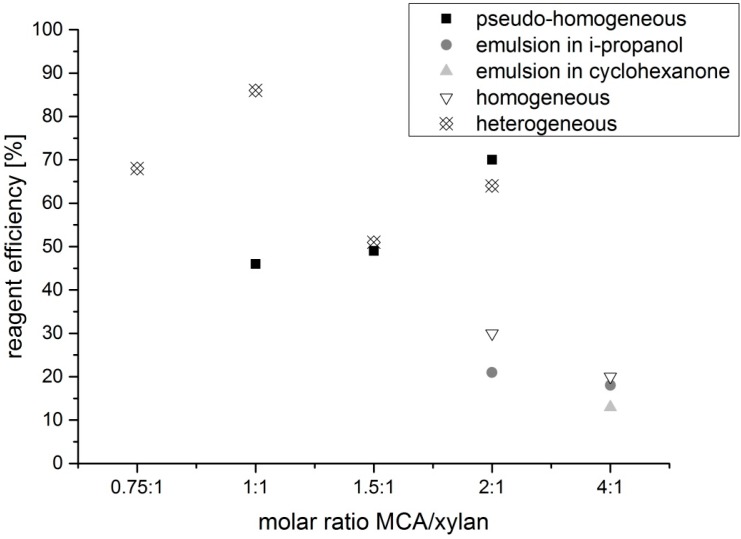
Reagent efficiency in dependence on the amount of used monochloroacetic acid (MCA) engaged for the various methods used.

Generally, the synthesis procedure of carboxymethyl xylan was chosen to be very similar to the ones commonly applied for the carboxymethylation of cellulose. First, the polysaccharide is alkalized with sodium hydroxide, followed by the reaction with chloroacetic acid (Figure 6). The synthesis of carboxymethyl xylan was investigated by varying the preparation method, molar ratio of reagents, temperature and reaction time (Table 2). High DS values of >1 were achieved by using heterogeneous reaction conditions in a stirring reactor or under pseudo-homogeneous conditions in a kneader (Figure 7). The reagent efficiencies under heterogeneous and under pseudo-homogeneous conditions were higher in comparison to the preparation under homogeneous conditions or in an emulsion (Figure 8). Under the latter two conditions, a higher excess of reagent was necessary to yield comparable DS values. Also, neither an increase of the reaction time from 24 to 72 h, nor of the temperature from 25 to 50 °C, showed any effect on the DS (CMX9 and CMX10). Nevertheless, we note that the homogeneously synthesized carboxymethyl xylans with comparatively low DS values of about 0.3 displayed good solubility in water. Amidst the products obtained by the various synthesis methods, the carboxymethyl xylans prepared under homogeneous conditions showed the best solubility, even though a slight turbidity was also observed (turbidity in the range from 30 to 60 NTU; nephelometric turbidity unit) at dry matter content of 2% (*w*/*w*). As expected, the DS increased with increasing of the ratio *n*_MCA_/*n*_xylan_. In the same order, the reaction efficiency decreased, while solubility was improved.

### 3.3. Dissolution Behaviour of the Xylan Ether

The dissolution behavior of the xylan derivatives was estimated by turbidity measurements. The aqueous solutions of the various xylan ethers showed a slight turbidity in contrast to aqueous solution of commercial cellulose ether, e.g., carboxymethyl and hydroxypropyl cellulose or starch ether. In the case of dihydroxypropyl xylan, the solubility depends on the synthesis method. The products prepared under pseudo-homogeneous conditions in a kneader are less soluble in comparison to the ones synthesized by the emulsion and homogeneous methods. This may be due to the lower DS value in the case of DHPX1 (Table 1), but also due to a more equal distribution of the substituents along the polymer chain in the case of a homogeneous synthesis. Also, in the case of carboxymethyl xylan, the water solubility of the homogeneously synthesized products was best, while the solubility of products with comparable DS values from the heterogeneous and pseudo-homogeneous syntheses did not differ from each other. Generally, the solubility was improved with increasing DS. In spite of high DS values of >1 of the prepared carboxymethyl xylans, totally transparent aqueous solutions were not obtained. We presume that the side chains of the pure xylan (e.g., arabino, glucuronic or rhamnose units), and the formation of small gel particles, are responsible for this effect. Also, the distribution of the carboxymethyl groups along the chain influenced the solubility of the products. As it is known for carboxymethyl cellulose with identical average DS values, products synthesized under homogeneous conditions show better solubility in comparison to the heterogeneously prepared ones [26]. This effect was also observed for the carboxymethylated xylans. Similarly, turbidity was also observed for dihydroxypropyl xylan solutions in water. Nevertheless, we note that nearly all xylan ethers prepared were soluble in water, and that no precipitation was detected after extended storage over months or after centrifugation. The turbidity declined slightly after heating the xylan ether solution, and did not change after subsequently cooling the samples. Temperature-induced phase separation with a lower or upper critical solution temperature (LCST or UCST), as frequently encountered for synthetic water-soluble polymers bearing hydroxyl groups [27,28,29,30,31], was not observed. Slight turbidity was also detected in alkaline or acidic solutions. We noted the carboxymethyl xylan solution showed yellow color and an increase of viscosity after increasing the pH.

**Figure 9:**
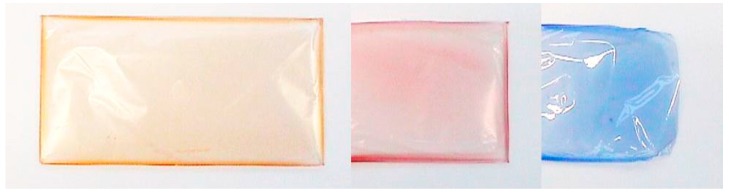
Cold water soluble films of dyed carboxymethylated xylan, cast from aqueous solutions.

Nevertheless, the prepared carboxymethyl xylans were well dispersible in water. Also, clear and transparent xylan ether films could be prepared from aqueous dispersion. When a dye was added to the dispersion, homogeneously colored clear films were obtained (Figure 9). 

These films can be easily redissolved by cold water. Hence, a water soluble film-forming material based on a biopolymer was obtained without any additives, such as plasticizers, etc. Such water soluble films or coating materials are useful for pharmaceutical, cosmetic, or other applications. Further investigations regarding the properties of these films are in progress.

## 4. Conclusions

Xylan which was isolated from oat spelts and its derivatives are attractive biobased polymers, and water soluble polymers can be generated by incorporation of functional ether groups. Thus, xylan ethers can be a low-cost alternative to the use of low viscous cellulose ethers. 

Two different water soluble xylan ethers—one ionic and one non-ionic—were successfully prepared by using various synthesis methods in this study. In particular, novel hemicellulose ethers were synthesized by using epoxypropanol as a reagent. For the synthesis of the xylan ethers, heterogeneous, pseudo-homogeneous, and homogeneous preparation methods were used. These synthesis methods, especially the heterogeneous ones, are typical for the industrial production of the analogous functional cellulose ethers. When preparing dihydroxypropyl xylan, no significant influence of the synthesis method on the DS was found. In contrast, clear differences were observed concerning the resulting DS values in the case of carboxymethyl xylan in dependence on the synthesis method. Carboxymethyl xylans with the highest DS were obtained by using kneader or heterogeneous methods. Xylan ethers with DS values of >1 could be synthesized in both cases. These xylan ethers showed interesting dissolution behavior. In spite of the lack of the primary OH-group in comparison to cellulose or starch, the synthesized xylan ethers were soluble in water, and produced freely flowing aqueous solutions. Moreover, stable, transparent, and dyeable biobased polymer films could be prepared from carboxymethyl xylans.

## Figures and Tables

**Table 1:** Results of synthesis of dihydroxypropyl xylan, dependant on the method (EProH = 2,3-epoxy-1-propanol).

Sample	*n*_EPrOH_/*n*_xylan_	*n*_NaOH_/*n*_xylan_	DS	Method
DHPX1	1:1	0.3:1	0.40	pseudo-homogeneous, 60 °C, 4 h
DHPX2	2:1	0.23:1	0.90	pseudo-homogeneous, 60 °C, 4 h
DHPX3	2:1	1:1	0.90	pseudo-homogeneous, 60 °C, 4 h
DHPX4	1:1	2.05:1	0.70	emulsion, i-propanol, 25 °C, 18 h
DHPX5	2:1	2.05:1	0.80	emulsion, i-propanol, 25 °C, 18 h
DHPX6	4:1	2.05:1	0.85	emulsion, i-propanol, 25 °C, 18 h
DHPX7	2:1	2.05:1	0.77	emulsion, cyclohexanone, 25 °C, 18 h
DHPX8	10:1	2.05:1	0.70	emulsion, cyclohexanone, 25 °C, 18 h
DHPX9	2:1	1.2:1	0.85	homogeneous, 25 °C, 24 h
DHPX10	4:1	2.05:1	0.88	homogeneous, 25 °C, 24 h
DHPX11	4:1	2.05:1	0.93	homogeneous, 25 °C, 95 h
DHPX12	10:1	2.05:1	1.22	homogeneous, 25 °C, 95 h

**Table 2:** Effect of the synthetic procedure on the synthesis of carboxymethyl xylan.

Sample	*n*_MCA_/*n*_xylan_	*n*_NaOH_/*n*_xylan_	DS	Method
CMX1	1:1	4.9:1	0.46	pseudo-homogeneous, 70 °C, 3 h
CMX2	1.5:1	4.7:1	0.73	pseudo-homogeneous, 70 °C, 4 h
CMX3	2:1	9.25:1	1.40	pseudo-homogeneous, 70 °C, 4 h
CMX4	2:1	9.25:1	0.42	emulsion, i-propanol, 60 °C, 4 h
CMX5	4.2:1	9.25:1	0.73	emulsion, i-propanol, 60 °C, 4 h
CMX6	4.2:1	9.25:1	0.55	emulsion, cyclohexanone, 60 °C, 4 h
CMX7	2:1	4.4:1	0.60	homogeneous, 50 °C, 3.5 h
CMX8	2:1	4.4:1	0.41	homogeneous, 50 °C, 8 h
CMX9	2:1	4.4:1	0.30	homogeneous, 25 °C, 24 h
CMX10	2:1	4.4:1	0.29	homogeneous, 50 °C, 72 h
CMX11	4.2:1	9.25:1	0.86	homogeneous, 50 °C, 3.5 h
CMX12	0.75:1	0.9:1	0.51	heterogeneous, 50 °C, 4 h
CMX13	1:1	2.2:1	0.86	heterogeneous, 50 °C, 4 h
CMX14	1.5:1	2.2:1	0.76	heterogeneous, 50 °C, 4 h
CMX15	2:1	4.4:1	1.28	heterogeneous, 50 °C, 4 h
